# Improving access to psilocybin-assisted therapy: barriers, challenges, and recommendations

**DOI:** 10.3389/fpubh.2026.1767210

**Published:** 2026-01-29

**Authors:** Vivian W. L. Tsang, Camille Roney, Pamela Kryskow, Shannon Dames

**Affiliations:** 1 Department of Psychiatry, Faculty of Medicine, University of British Columbia, Vancouver, BC, Canada; 2 Evidence Based Healthcare, University of Oxford, Oxford, United Kingdom; 3 Undergraduate Medical Program, Faculty of Medicine, University of British Columbia, Vancouver, BC, Canada; 4 Department of Family Medicine, Faculty of Medicine, University of British Columbia, Vancouver, BC, Canada; 5 Health and Human Services, Vancouver Island University, Nanaimo, BC, Canada

**Keywords:** end-of-life care, health policy, palliative care, policy, psilocybin, psychedelics, public health

## An introduction to psilocybin-assisted therapy

Psilocybin-assisted therapy (PAT) is an emerging intervention that combines the administration of psilocybin with structured psychological support to address profound psychological, emotional, and existential distress. Central to this model is the recognition that many forms of suffering—particularly at end of life—are relational in nature and require relational forms of healing. It is our belief that the relational wounds that keep us from connection can really only be healed in relationship with other humans (1). This emphasis on relational healing lies at the core of PAT, a treatment model designed to address profound psychological distress through both pharmacological and human-centered care.

Located in Nanaimo, British Columbia, Roots to Thrive is currently the only multidisciplinary non-profit healthcare practice in Canada legally offering group PAT (11–13, 18, 20). The program primarily serves individuals facing terminal illness, many of whom seek relief from the psychological symptoms that often accompany end-of-life diagnoses. Anxiety, depression, unresolved trauma, and existential distress can be as consequential as physical symptoms, significantly impacting treatment adherence, quality of life, and overall wellbeing (2). Among terminally ill patients, psychological distress is associated with decreased life satisfaction, withdrawal from meaningful relationships, increased suicidality, and even earlier death (3).

Despite strong evidence supporting PAT's effectiveness, demand for this transformative therapy far exceeds accessibility. Canada's Special Access Program (SAP) is a regulatory mechanism that allows healthcare practitioners to request access to drugs that are not otherwise approved for sale when conventional treatments have failed, are unsuitable, or are unavailable (15–17). Under the SAP, psilocybin may be authorized on a case-by-case basis for patients with serious or life-threatening conditions, provided there is a clear medical rationale and appropriate clinical oversight. Between 2022 and late 2024, there were 471 total SAP applications submitted to access psilocybin or MDMA for therapeutic use in Canada. Of these, only about 318 were approved. Many individuals die before ever receiving relief. This advocacy piece highlights existing barriers and challenges for patients.

## What psilocybin-assisted therapy looks like in practice

The PAT program offered through Roots to Thrive consists of eight to ten small-group therapy sessions. One of these sessions includes the administration of psilocybin via oral route of administration (4). The remaining sessions focus on preparation, integration, and relational support delivered through a community-of-practice (CoP) model. In this body of work, a CoP is conceptualized as a structured, collaborative clinical model in which practitioners engaged in psychedelic- and ketamine-assisted therapies participate in ongoing collective learning, reflective practice, and shared clinical responsibility. This model is particularly well suited to novel and highly regulated therapeutic contexts, where clinical complexity and ethical considerations necessitate collective expertise and accountability. Although we do offer individual PAT, the group structure intentionally situates the psychedelic experience within a broader therapeutic and communal framework recognizing the group as part of the healing process rather than treating it as a standalone intervention.

The program is facilitated by a multidisciplinary team that may include registered psychotherapists, nurses, physicians, and Indigenous Knowledge Keepers. All providers are trained through their respective colleges and also through specific psychedelic assisted therapy training programs in Canada (5). Central to the model are unconditional positive regard, emotional safety, and community-based healing. These relational elements are not ancillary; they are foundational to the therapeutic outcomes reported by participants.

From a clinical standpoint, PAT has demonstrated remarkable efficacy and safety. Research shows that PAT produces rapid and sustained antidepressant effects among terminally ill individuals experiencing mood or anxiety disorders (3, 6). Approximately 80% of end-stage cancer patients who receive PAT experience sustained clinical symptom improvement—outcomes that stand in stark contrast to conventional antidepressant treatments, which often perform little better than placebo in this population (3, 7).

Concerns about safety are frequently raised in policy discussions; however, psilocybin has a well-established safety profile. The Registry of Toxic Effects of Chemical Substances (RTECS) has assigned psilocybin a more favorable safety profile than aspirin (8). Importantly, PAT is not intended to replace antidepressants or psychotherapy outright. Instead, psilocybin is used as a catalyst to enhance therapeutic effectiveness for individuals who have not experienced relief through other interventions.

## Barriers to care

Despite growing evidence and patient demand, access to PAT remains profoundly restricted. For individuals with a terminal diagnosis, the process of obtaining legal access is lengthy and burdensome. Patients must secure a referral from a physician or nurse practitioner, complete extensive medical intake assessments, apply to Health Canada's SAP, attend multiple education and integration sessions, and then wait for placement in a clinical trial or approved treatment cohort. For patients whose physical or cognitive capacity declines as their illness progresses, these delays can be devastating. As a result, the Roots to Thrive team spends countless hours advocating for expedited access on behalf of patients whose time is rapidly diminishing.

Patients also reported significant emotional and logistical barriers. Many—particularly women—expressed fear of judgment when discussing PAT with their physicians. Others cited anxiety about long wait times, transportation challenges, financial strain, and the difficulty of coordinating PAT alongside complex treatment regimens for terminal illness.

Geography further compounds these challenges. Because Health Canada has approved only a small number of PAT providers, most patients must either travel to Nanaimo for the full eight-to-ten-week program or attempt to coordinate care remotely with their primary provider physically present. For many, this is simply not feasible. As one patient, Thomas Hartle, explains, “I have to put chemotherapy on pause just to go out there. So I'm compromising my health, and I'm emptying my bank account.” For individuals with limited mobility or those requiring assistance from a caregiver, travel is often impossible.

Systemic inequities also disproportionately affect Black, Indigenous, and People of Color (BIPOC). Data from the OurCare National Survey indicate that individuals who self-identify as racialized are less likely to have a family doctor or nurse practitioner, limiting their ability to even initiate the SAP process (9). Dr. Gail Peekeekoot, Medicine Administration Nursing Lead and Facilitator at Roots to Thrive, emphasizes that cultural traditions integral to psychedelic healing are often lost within the current medicalized framework (19). For Indigenous patients without access to legal providers who share their language, values, or cultural worldview, she explains, “We lose ceremony, we lose the sacred.”

## Access through the Special Access Program (SAP)

Conventional palliative care teams—already stretched thin—often lack the resources to adequately address the psychological and existential distress experienced at end of life (2). Ironically, these are precisely the areas where PAT has shown the most promise. Despite growing clinical evidence and patient demand, access to PAT in Canada remains tightly restricted due to its classification as a Schedule III substance under the Controlled Drugs and Substances Act. Legal access is primarily mediated through Health Canada's SAP, a pathway designed for exceptional circumstances but increasingly relied upon for routine compassionate use in palliative contexts (10).

Good healthcare should not be political. Currently, only physicians or nurse practitioners may apply to the SAP on a patient's behalf. The SAP application is an extensive eight-page document requiring clinicians to identify an approved manufacturer, justify psilocybin use with comprehensive clinical evidence, document all previous failed treatments, oversee clinical trial participation if applicable, remain physically present during the entire drug session, and submit detailed post-treatment reports (Notice to Stakeholders, 2022; 2023). These requirements are unrealistic for most primary care providers and fail to recognize the expertise of non-physician practitioners already trained in PAT delivery. This step moves the governance and use of psilocybin from a medical decision to a parliamentary decision.

In response to the volume of SAP applications submitted by Roots to Thrive, Health Canada requested in Fall 2022 that the organization transition entirely to a clinical trial framework. However, we argue that clinical trials are ill-suited to meet the urgent needs of patients who are terminally ill. Prioritizing expensive clinical trials researching new aspects of PAT should not be a barrier to treatment illnesses with sufficient evidence to already be known to benefit from PAT.

In the meantime, many individuals who sought treatment have either died or continue to wait in distress for what they consider their last hope for relief. As world-renowned mycologist Paul Stamets notes, such regulatory delays raise serious ethical concerns, describing the approach as “not only academically naive, but immoral (14).” While subjective, this critique reflects a broader ethical unease shared by clinicians working with dying patients.

## Moral distress among patients and providers

For the Roots to Thrive team, advocating for access to PAT has come at a significant emotional cost. Thousands of hours of unpaid clinician labor have been dedicated to navigating regulatory barriers, often only to receive delays or denials after patients have already passed away. “These people are dying. They are in distress,” says Dr. Dames. “What if it was your mother or your child, or your life? Why wouldn't you want the best for them like this?”

Physicians, nurses, facilitators, and Knowledge Keepers described profound moral and ethical distress as they watched patients deteriorate while awaiting approval. “We won't have treated anyone [with PAT] for an entire year because we've been trying to figure out how a non-profit does a clinical trial in order to give people care while they're dying,” one provider shared.

Patients echoed this distress. Thomas waited 511 days for Health Canada to deny his reapplication for continued PAT access, noting, “I could get access to the MAID system in 73 h.” Reflecting on regulatory priorities, he added, “If you were looking at where Health Canada is placing their emphasis, it's ‘end your life, rather than look for a better quality of life with the time you have left.”'

These experiences directly contradict Health Canada's own SAP Guidance Document, which states that most requests are processed within one working day and that personalized support is provided for end-of-life cases—services none of the surveyed providers reported receiving (10, 15–17).

As access remains restricted, some patients turn to unregulated underground providers. Many of these practitioners lack adequate training, insurance, or oversight. Dr. Peekeekoot adds that stigma and legal barriers push individuals to self-administer psychedelic mushrooms in isolation, simply “hoping for the best” to ease their suffering.

## Where do we go from here?

As psychedelic-assisted therapies move closer to mainstream clinical acceptance, attention must shift from questions of efficacy to questions of access, equity, and implementation. Without regulatory reform, the public health benefits of PAT will remain inaccessible to those most likely to benefit. Although Health Canada has made incremental progress toward acknowledging PAT's role in palliative care, substantial reform is still required. In addition to existing efforts, the following steps could meaningfully reduce barriers to care:

1. Establish a streamlined referral pathway allowing primary care providers to refer directly to approved and accredited PAT providers.
2. Implement a unique dedicated SAP fast-track for end-of-life patients with guaranteed review timelines.
3. Develop a formal provincial billing framework for PAT.
4. Expand practitioner education on psychedelic-assisted therapies within medical and nursing curricula.
5. Introduce public insurance coverage (e.g., medical services plan) for PAT in palliative and treatment-resistant contexts.
6. Reform legislative frameworks to reflect current evidence and reduce stigma associated with psychedelic medicines.
7. Mandate integration and family-inclusive support as standard components of PAT.
8. Partner with non-profit organizations to deliver community-based models of care.
9. Support Indigenous-led psychedelic healing pathways grounded in cultural safety and sovereignty.
10. Fund implementation research to establish standardized dosing, safety, and delivery guidelines.

As Dr. Dames emphasizes, “We're all one medical team. We're all tired and really just trying to help the same people.” For Thomas, the impact of PAT was profound. Reflecting on his experience, he shares, “It's the difference between actively living and actively dying. I wish other people would be able to experience that.”
